# Efficacy of a Novel Therapeutic, Based on Natural Ingredients and Probiotics, in a Murine Model of Multiple Food Intolerance and Maldigestion

**DOI:** 10.3390/nu14112251

**Published:** 2022-05-27

**Authors:** Alessio Ardizzone, Marika Lanza, Giovanna Casili, Michela Campolo, Irene Paterniti, Salvatore Cuzzocrea, Emanuela Esposito

**Affiliations:** Department of Chemical, Biological, Pharmaceutical and Environmental Sciences, University of Messina, Viale Ferdinando Stagno D’Alcontres, 98166 Messina, Italy; aleardizzone@unime.it (A.A.); mlanza@unime.it (M.L.); gcasili@unime.it (G.C.); campolom@unime.it (M.C.); ipaterniti@unime.it (I.P.); salvator@unime.it (S.C.)

**Keywords:** food intolerances (FIs), maldigestion, high-carbohydrate diet (HCD), high-fat diet (HFD), high-fructose diet (HFrD), polysaccharides, pea protein, grape seed extract, barrier integrity

## Abstract

Patients with hypersensitive gut mucosa often suffer from food intolerances (FIs) associated with an inadequate gastrointestinal function that affects 15–20% of the population. Current treatments involve elimination diets, but require careful control, are difficult to maintain long-term, and diagnosis remains challenging. This study aims to evaluate the beneficial effects of a novel therapeutic of natural (NTN) origin containing food-grade polysaccharides, proteins, and grape seed extract to restore intestinal function in a murine model of fructose, carbohydrate, and fat intolerances. All experiments were conducted in four-week-old male CD1 mice. To induce FIs, mice were fed with either a high-carbohydrate diet (HCD), high-fat diet (HFD), or high-fructose diet (HFrD), respectively. After two weeks of treatment, several parameters and endpoints were evaluated such as food and water intake, body weight, histological score in several organs, gut permeability, intestinal epithelial integrity, and biochemical endpoints. Our results demonstrated that the therapeutic agent significantly restored gut barrier integrity and permeability compromised by every FIs induction. Restoration of intestinal function by NTN treatment has consequently improved tissue damage in several functional organs involved in the diagnostic of each intolerance such as the pancreas for HCD and liver for HFD and HFrD. Taken together, our results support NTN as a promising natural option in the non-pharmacological strategy for the recovery of intestinal dysregulation, supporting the well-being of the gastrointestinal tract.

## 1. Introduction

Food intolerances (FIs) are non-immunological disorders that occur after consuming a specific food or one of its components [1]. FIs are very common, affecting about 15–20% of the population in industrialized countries [2]; despite their wide diffusion, their etiopathogenesis is not yet fully known. It has been demonstrated that the excessive ingestion of a certain food predisposes the patient to develop a sensitization towards it [3].

In this context, given the greater consumption of industrial food in Western countries, FIs and maldigestion have increased exponentially in recent years [4]. The diet is an important factor that determines the well-being of the individual; relatedly, it was proven that changes in lifestyle and in the dietary composition constitute a predisposing factor to the development of various diseases [5]. In particular, high carbohydrate intake, consisting mainly of food with a high glycemic index, has harmful metabolic effects [6] that may lead to the development of diseases such as gastrointestinal disorders and metabolic syndrome, and may increase the risk of cardiovascular disease (CVD) as well as to diabetes mellitus [7,8]. Similarly, unhealthy fatty meals with a high content of saturated fatty acids represent a predisposing factor to obesity, CVD [9], gastrointestinal disease [10], and dyslipidemia [11], also reducing intestinal barrier function [12]. In addition, a large body of evidence warns of the risks of excessive fructose consumption. Fructose is a molecule classifiable as a 6-carbon monosaccharide, which is present naturally in a wide range of foods such as fruits, vegetables and honey [13]. In the last 40 years, its use as a food sweetener has grown exponentially [14]. Consequently, its higher consumption in the population has led to an increase of fructose malabsorption and intolerance, which has often been associated with unexplained bloating, belching, distension, gas, abdominal pain or diarrhea [15]. Based on what was previously disclosed, high sugar or lipid intakes can worsen the patient’s quality of life, promoting metabolic changes and dysregulating the homeostasis of the gastrointestinal tract. Certainly, in the multifaceted etiology of these food disorders, intestinal barrier disruption has a significant role. The alteration of the intestinal barrier following a non-balanced diet affects the metabolic machinery responsible for digestion and the absorption of nutrients [16]. Thus, hypersensitivity of the intestinal mucosa leads to a partial or total loss of the ability to digest, altering nutrients sensing in the digestive tract [17]; in which also epithelial tight junction (TJs) loss plays a key role [18].

Hence, it is reasonable to assume that FIs patients need a solution to restore the integrity of the intestinal barrier. Currently, elimination diets remain the accepted way of dealing with FIs, but they are not easy to follow and often lead to nutritional deficiencies. For these reasons, the discovery of other therapeutic options is necessary. Non-pharmacological treatment options are of great support to traditional therapy, improving health status and proving to be safe and effective in managing the symptoms of various intestinal disorders and FIs [19,20].

On this basis, this study aimed to evaluate the beneficial effects of a novel therapeutic of natural origin (NTN) for the treatment of FIs. NTN contains probiotics and natural compounds that are considered a remarkably and effective nonpharmacological option in counteracting gut dysbiosis and intestinal injury. Indeed, *Lactobacillus acidophilus* and *Lactobacillus reuteri* are gram positive bacteria which provide a valid support in the equilibrium of intestinal microflora, normalizing the passage of stool as well as stool consistency in subjects suffering from intestinal disorders [21]. Acacia and Pea protein are natural compounds exercising an emollient and soothing action in the digestive tract thanks to their high fiber content [22,23]. In particular, Acacia, mainly composed of complex polysaccharides, resists digestion in the upper gastrointestinal tract, thus reaching the large intestine in which it can induce an increase in *Bifidobacterium* spp. [22]. Similarly, Pea protein modulates intestinal bacteria activities [23]; thus, both natural compounds are able to improve gut mucosal barrier and gut homeostasis. β-galactosidase, thanks to its enzymatic activity, are helpful in the case of galactose-containing carbohydrate intolerances, as shown by pre-clinical and clinical studies [24]. NTN also contains grape seeds extract, a suitable source of proanthocyanidins with valuable antioxidative properties. This natural compound has been revealed to improve intestinal health by reverting plasma bacterial endotoxins to basal levels [25]. Therefore, considering the beneficial properties of the aforementioned compounds in providing intestinal relief, we assessed NTN in multiple murine models of FIs: carbohydrate, lipid, and fructose.

## 2. Materials and Methods

### 2.1. Materials

Standard diet (SD), high-carbohydrate diet (HCD), high-fat diet (HFD) or high-fructose diet (HFrD) were purchased from Envigo (Milan, Italy). The product containing *Acacia senegal* (L.) Willd, tyndallized *L. acidophilus*, tyndallized *L. reuteri*, Pea protein, Grape seed extract and β-galactosidase was kindly provided by DEVINTEC SAGL (Lugano, Switzerland). The human doses shown in Table 1 were converted to mouse doses based on the body surface formulation [26]. The total dose of NTN administered to each mouse was: 37 mg/kg. Unless otherwise stated, all compounds employed in this study were obtained from Sigma-Aldrich (Poole, UK). For oral administration, NTN was dissolved in saline and given to the mice three times a day by oral gavage.

### 2.2. Animals

Male CD1 mice (Envigo, Milan, Italy) at four weeks of age were used. Mice were housed in a controlled environment (22 ± 2 °C, 55 ± 15% relative humidity, 12 h light/dark cycle), with food and water ad libitum. Before this study, the animals were kept in a quarantine area for one week. During this period, they were observed daily. In addition, a numbered tag placed through the edge of the right ear identified the animals selected for the study. Animal experiments were in compliance with Italian regulations on the protection of animals used for experimental and other scientific purposes (DM 116192), as well as EU regulations (OJ of EC L 358/1 12/18/1986) and ARRIVE guidelines.

### 2.3. Experimental Design

At the end of the quarantine week, the animals were carefully examined to evaluate their suitability for the study and randomly divided into several experimental groups to induce the specific FIs.

#### 2.3.1. HCD Induction

For the induction of carbohydrate intolerance, mice were fed *ad libitum* with an HCD (Table 2) diet for five weeks [27]. Control animals were fed *ad libitum* with an SD.

Experimental groups

Group 1 SD; mice were fed with a SD plus vehicle for three weeks (*n* = 4);
Group 2 SD + NTN mice were fed with a SD for three weeks plus oral administration of NTN for the next two weeks (*n* = 8);
Group 3 HCD; mice were fed with an HCD for three weeks plus oral administration of vehicle for the next two weeks (*n* = 8);
Group 4 HCD + NTN; mice were fed with an HCD for three weeks plus oral administration of NTN for the next two weeks (*n* = 8);

#### 2.3.2. HFD Induction

For the induction of lipid intolerance, mice were fed an HFD ad libitum (60% kcal derived from fat) for 14 weeks [28]. Control animals were fed an SD ad libitum.

Experimental groups

Group 1 SD; mice were fed with an SD plus vehicle for 14 weeks (*n* = 4);
Group 2 SD + NTN; mice were fed with an SD for 12 weeks plus oral administration of NTN for the next two weeks (*n* = 8);
Group 3 HFD; mice were fed with an HFD for 12 weeks plus oral administration of vehicle for the next two weeks (*n* = 8);
Group 4 HFD + NTN; mice were fed with an HCD for 12 weeks plus oral administration of NTN for the next two weeks (*n* = 8);

#### 2.3.3. HFrD Induction

For the induction of fructose intolerance, Mice were fed ad libitum an HFrD for 15 weeks [29,30] (30% in drinking water). Control animals were fed an SD ad libitum.

Experimental groups

**Figure 1:**
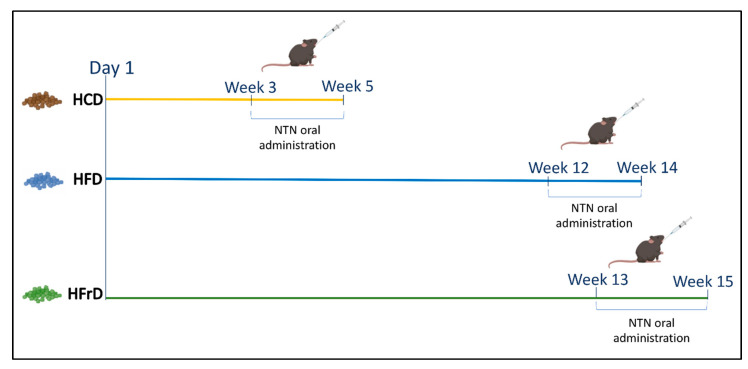
Timeline of FIs. The figure summarizes the timing of each experimental model. Five weeks for carbohydrate intolerance (the administration of NTN was conducted in the last two weeks); 14 weeks for lipid intolerance (the administration of NTN was carried out in the last two weeks); 15 weeks for fructose intolerance (the administration of NTN was carried out in the last two weeks).

Group 1 SD; mice were fed with an SD plus vehicle for 15 weeks (*n* = 4);
Group 2 SD + NTN; mice were fed with a SD for 13 weeks plus oral administration of NTN for the next two weeks (*n* = 8);
Group 3 HFrD; mice were fed with an HFrD for 13 weeks plus oral administration of vehicle for the next two weeks (*n* = 8);
Group 4 HFrD + NTN; mice were fed with an HFrD for 13 weeks plus oral administration of NTN for the next two weeks (*n* = 8);

At the end of the experiments, animals were sacrificed and tissues were surgically removed and processed for histological examinations and biochemical analyses. In addition, the blood of each mouse was collected for further biochemical assay. The timeline of every FI induction is summarized in Figure 1.

### 2.4. Histological Evaluations

Histological analyses were performed as previously described by Casili et al. [31] and reported below. Immediately after the sacrifice of the animals, samples were fixed in 10% (*w*/*v*) PBS-buffered formaldehyde solution at 25 °C for 24 h. After dehydration, samples were included in paraffin. Tissue sections of 5 μm were stained with Hematoxylin/Eosin (H&E, Bio-Optica, Milano, Italy) to evaluate histological alterations of pancreas, abdominal adipose and intestine tissues in HCD; liver and abdominal adipose tissues in HFD; liver and intestine tissues in HFrD. The results of histological examinations were displayed at 10× magnification (100 µm scale bar). All the histological analyses were performed in a blinded manner.

### 2.5. Immunohistochemical Localization of ZO-1 and Occludin

Immunohistochemical localization of TJs in HCD and HFrD tissues were done as previously described by Campolo et al. [32]. Slices were incubated at room temperature overnight with the following primary antibodies: anti-zonula occludens-1 (ZO-1) (Santa Cruz Biotechnology sc-33725, 1:100 in PBS, *v*/*v*) and anti-occludin (Santa Cruz Biotechnology sc-133256; 1:100 in PBS, *v*/*v*). After primary antibody incubation, sections were washed in PBS and incubated with secondary antibody (Santa Cruz Biotechnology, Dallas, TX, USA) for 1 h. The reaction was revealed by a chromogenic substrate (DAB), and counterstaining with Nuclear Fast Red (Bio Optica, Milan, Italy). For a graphic display of the densitometric analyses, the % of positive staining (DAB brown staining) was measured by computer-assisted color image analysis (Leica QWin V3, Newcastle, UK). The percentage area of immunoreactivity (determined by the number of positive pixels) was expressed as % of total tissue area (red staining) within five random fields at a 40× magnification. For immunohistochemistry, 20× (50 µm scale bar) and 40× (20 µm scale bar) were shown. Immunohistochemical studies were performed in a blinded fashion.

### 2.6. Gut Permeability

FITC-dextran was used to measure the intestinal permeability in HCD, HFD and HFrD animals.

Mice were fasted for 6 h, after which FITC-dextran was administered by gavage (500 mg/kg body weight, 125 mg/mL). Subsequently, 100 µL of blood was collected from the caudal vein after 1 h and 4 h. The blood was centrifuged at 12,000× *g* for 5 min at 4° C. The plasma concentration of dextran was measured with a microplate reader (Molecular Devices, Sunnyvale, CA, USA) with an excitation wavelength of 485 nm and an emission wavelength of 535 nm. The standard curve was created by diluting FITC-dextran in untreated plasma diluted with phosphate–buffered saline (1:1, *v*/*v*).

### 2.7. Plasma Insulin and Glucose Levels

In the HCD study, blood was collected from the tail vein of each mouse and subsequently centrifuged for 10 min, at 3000× *g*, 4 °C; plasma was stored at −20 °C for assay of insulin and glucose levels as previously reported [27,33]. Plasma glucose was measured spectrophotometrically using commercially available colorimetric kits (Aspen Laboratories Pvt. Ltd., New Delhi, India) and expressed as plasma glucose (mg/dL) levels. Plasma insulin was detected using an enzyme-linked immunoassay.

### 2.8. Analysis of Liver Weight

Hepatic steatosis was evaluated as previously described by Tao et al. [34]. At the end of HCD experiment, the weight of livers was measured through analytical balance (Bel engineering balance; Monza, Italy) to evaluate the effects of the high carbohydrate intake on lipid hepatic accumulation.

### 2.9. Quantification of NEFA and TG

A lipid tolerance test was performed based on Peterson et al. [28]. Fasted mice were IP-injected with 20% emulsified Intralipid (10 mL/g of body weight Sigma Aldrich), mimicking the sudden rise of plasma lipids in response to food intake. Sera was collected via tail bleed using a MicrovetteH CB 300 (Sarstedt) at 0, 1, 2, 3, and 5 h post-injection. Serum levels of non-esterified fatty acids (NEFA) and triglycerides were quantified using kits from Wako and Infinity Triglycerides, respectively.

### 2.10. Statistical Analysis

Experimental data are expressed as mean ± standard error of the mean (SEM) of *n* observations, in which *n* represents the number of animals studied. In the experiments involving histological evaluations, images are representative of at least three independent experiments. In order to reach the minimum number of mice required for every technique, an ANOVA (fixed effects, omnibus, one-way) was defined “a priori” with the G-power software. This statistical test supplies a professional method to analyze the sample size required to make the experiments.

Data analysis was performed with One-Way and Two-Way ANOVA followed by a Bonferroni post-hoc test for multiple comparisons. Only a *p*-value less than 0.05 was considered significant.

## 3. Results

### 3.1. Effects of NTN Administration on Body Weight, Food Intake, Pancreas Tissue Damage and Glucose-Insulin Levels in HCD Mice

The weight gain that occurs during NTN intake is due not to a physiological or metabolic consequence of monosaccharides or disaccharides, but to a modification of sugar intake resulting from an alteration in energy balance [35] that alters the hunger-satiety continuum, thus facilitating carbohydrate consumption in the absence of energy needs [36].

**Figure 2:**
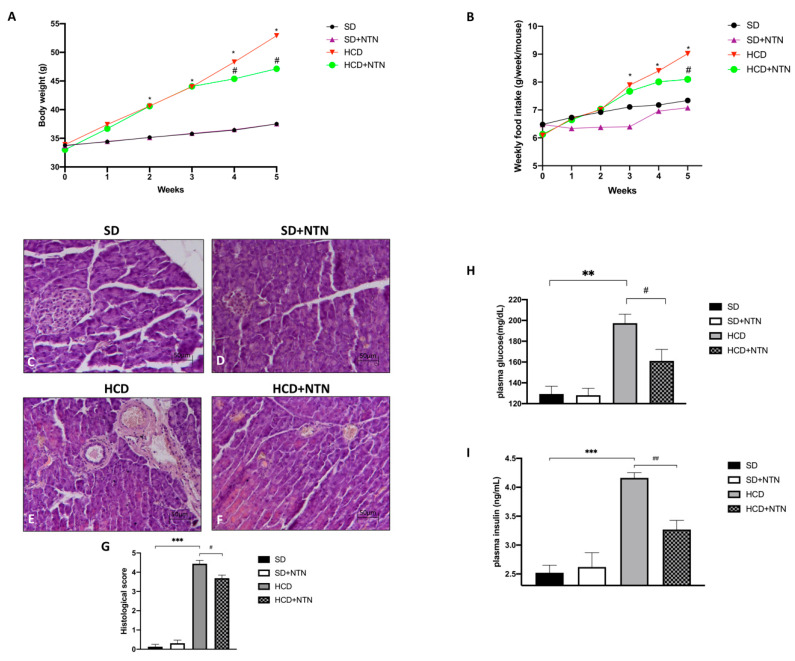
Effects of NTN on body weight, food intake, histological damage of the pancreas and glucose-insulin levels in HCD intolerant mice. A slight increase in body weight and food intake was detected in HCD-mice compared to the control group (**A**,**B**); NTN administration reduced both parameters in HCD mice (**A**,**B**). Extensive neutrophil infiltration and tissue damage were observed in mice fed with HCD (**E**,**G**) compared to SD and SD + NTN animals (**C**,**D**,**G**). Administration of NTN was able to significantly counteract the extent of tissue damage and neutrophil infiltration in HCD mice (**F**,**G**). NTN administration reduced both glucose and insulin levels (**H**,**I**). Data are representative of at least three independent experiments. Values are means ± SEM. One-Way and Two-Way ANOVA test. * *p* < 0.05 vs. SD; ** *p* < 0.01 vs. SD; *** *p* < 0.001 vs. SD; # *p* < 0.05 vs. HCD; ## *p* < 0.01 vs. HCD.

After five weeks of HCD, the mice showed a moderate weight gain and an increased food intake compared to the control group (Figure 2A,B); NTN treatment was able to reduce body weight already after one week of treatment (week 4 in the graph Figure 2A) in mice fed with HCD as well as to restrain carbohydrate consumption (Figure 2A,B).

A high dietary carbohydrate intake results in elevated circulating glucose levels and hyperinsulinemia [37] as well as pancreatic β cell dysfunction, thus leading to poor management of the glycemic load [38]. In relation to this, we analyzed the tissue integrity of the pancreas by H&E staining to evaluate the morphological changes after HCD.

A significant increase of tissue damage was found in the pancreas of HCD mice, accompanied by moderate hyperplasia of the islet of Langerhans and neutrophilic infiltration (Figure 2E, histological score Figure 2G) compared to the control group (Figure 2C, histological score Figure 2G). However, NTN administration significantly improved the pancreas tissue architecture (Figure 2F, histological score Figure 2G), a feature correlating also with the better management of glycemic and insulin parameters, a notable feature of carbohydrate intolerance. In fact, following the HCD diet, we assisted in a marked increase in both glucose (Figure 2H) and insulin (Figure 2I) plasma levels compared to SD; conversely, the two-week treatment with NTN considerably reduced both parameters.

### 3.2. Effects of NTN Administration on Abdominal Adipose Tissue Damage and Steatosis in HCD Mice

**Figure 3:**
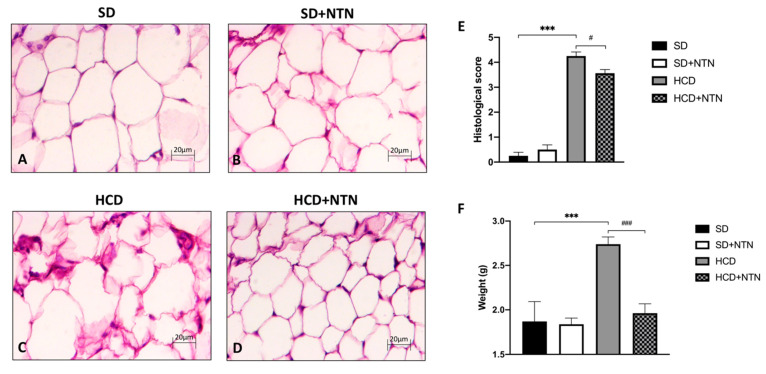
Effect of NTN on adipose abdominal tissue and liver in HCD mice. HCD mice showed a significant tissue injury in adipose tissue (**C**,**E**); to the contrary, SD and SD + NTN mice showed no tissue damage (**A**,**B**,**E**). NTN restored physiological parameters, thus reducing neutrophil infiltration and adipocytes size (**D**,**E**). In addition, NTN was able to decrease liver weight (**F**). Data are representative of at least three independent experiments. Values are means ± SEM. One-Way ANOVA test. *** *p* < 0.001 vs. SD; # *p* < 0.05 vs. HCD; ### *p* < 0.001 vs. HCD.

Histopathological evaluation of carbohydrate intolerant mice displayed a significant increase of tissue damage and neutrophil infiltration in white abdominal adipose tissue (Figure 3C, histological score Figure 3E) compared to SD mice (Figure 3A, histological score Figure 3E); NTN supplementation appreciably restored the architecture of the abdominal adipose tissue (Figure 3D, histological score Figure 3E). A decrease in adipose content was also found in the liver; in fact, the administration of NTN was able to significantly reduce hepatic steatosis caused by excessive calorie intake (Figure 3F).

### 3.3. Effects of NTN Administration on Intestinal Tissue Damage and Permeability

**Figure 4:**
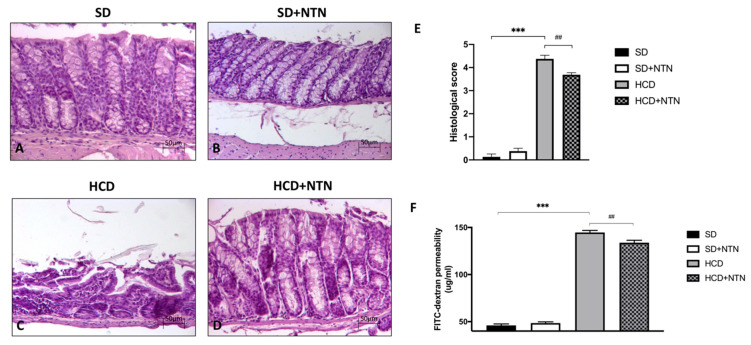
Effects of NTN on intestinal tissue damage and permeability in HCD mice. Neutrophil infiltration and tissue damage was observed in mice fed with HCD (**C**,**E**) compared to SD and SD + NTN animals (**A**,**B**,**E**). Administration of NTN was able to significantly counteract the extent of intestinal tissue damage and neutrophil infiltration in HCD mice (**D**,**E**). FITC-dextran permeability assay of HCD mice jejunum exposed a marked increase of intestinal permeability; NTN exerted an important protective barrier effect (**F**). Data are representative of at least three independent experiments. Values are means ± SEM. One-Way ANOVA test. *** *p* < 0.001 vs. SD; ## *p* < 0.01 vs. HCD.

Excessive consumption of carbohydrates leads to intestinal disorders characterized by intestinal dysregulated morphology, accompanied by high intestinal permeability and loss of tissue epithelial integrity [39]. A significant increase in intestinal tissue damage and neutrophil infiltration was observed in carbohydrate intolerant mice (Figure 4C, histological score Figure 4E) compared to the control group (Figure 4A, histological score Figure 4E). NTN administration significantly improved tissue architecture of the intestine counteracting the extent of intestinal tissue damage and neutrophil infiltration due to HCD (Figure 4D, histological score Figure 4E).

Furthermore, to evaluate the barrier protective properties of NTN we assessed gut permeability with a Transelectrical Epithelial Resistance (TEER) test.

A marked increase in gut permeability was observed in mice fed with HCD compared to mice fed with SD (Figure 4F). NTN, after two weeks of treatment, significantly reduced the increase in the paracellular FITC-dextran flux induced by HCD, proving to be a good regulator of gut permeability (Figure 4F).

### 3.4. Effects of NTN Administration on Intestine Epithelial Integrity in HCD Mice

TJs are multiprotein intercellular junctions adjacent to the apical ends of the paracellular spaces [40]. The main components are ZO-1 and Occludin, which among their main functions, regulate cellular permeability and barrier intestinal function. Consequently, their dysregulation is often associated with bowel disease.

**Figure 5:**
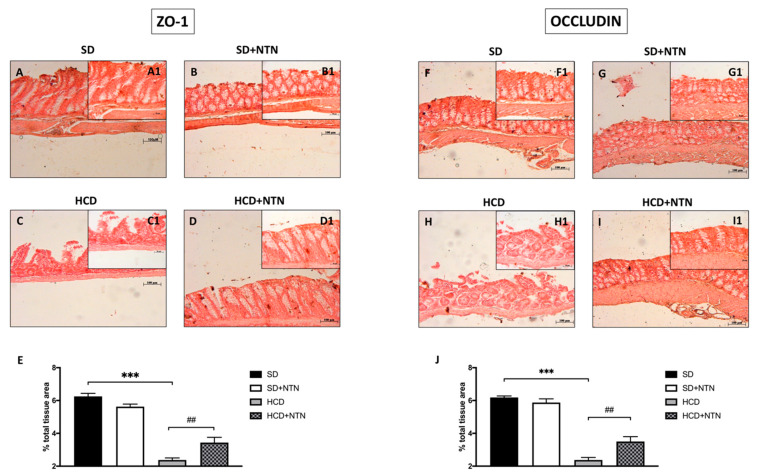
Effects of NTN administration on intestine epithelial integrity in HCD mice. A high percentage in the expression of ZO-1 (**A**,**B**,**E**) and Occludin (**F**,**G**,**J**) were found in intestinal tissues of SD mice, conversely HCD decreased such expressions (**C**,**E**,**H**,**J**). NTN has appreciably restored the levels of ZO-1 (**D**,**E**) and Occludin (**I**,**J**). Data are representative of at least three independent experiments. Values are means ± SEM. One-Way ANOVA test. *** *p* < 0.001 vs. SD; ## *p* < 0.01 vs. HCD.

To evaluate the beneficial effect of NTN on intestinal epithelial integrity, we estimated ZO-1 and Occludin expression through immunohistochemical analysis. Mice fed with HCD displayed a significant decrease in ZO-1 (Figure 5C, histological score Figure 5E) and Occludin expressions (Figure 5H, histological score Figure 5J) compared to control mice (Figure 5A and Figure 5F respectively, histological score Figure 5E and Figure 5J respectively). The two weeks of treatment with NTN notably improved the integrity of the intestinal barrier, promoting the increase in the expression of ZO-1 (Figure 5D, histological score Figure 5E) and Occludin (Figure 5I, histological score Figure 5J).

### 3.5. Effects of NTN Administration on Body Weight, Food Intake, Liver Tissue Damage, Lipid Tolerance Parameters and Gut Permeability in HFD Mice

**Figure 6:**
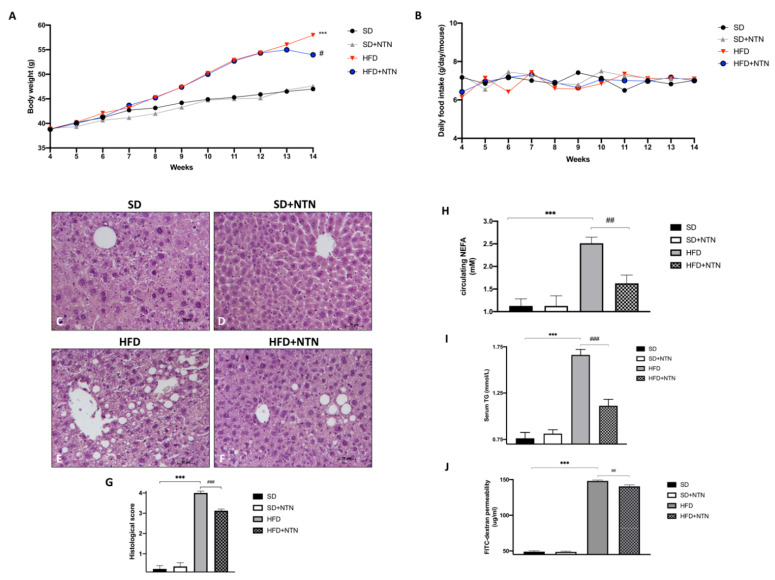
Effects of NTN on liver tissue, fat mobilization and gut permeability in HFD mice. HFD-fed mice showed a significant increase in body weight compared to the sham group (**A**); NTN considerably decreased weight gain (**A**). No significant differences were detected in mice food intake (**B**). Significant hydropic degeneration and steatosis were observed in mice fed with HFD (**E**,**G**) compared to SD and SD + NTN animals (**C**,**D**,**G**). Administration of NTN was able to significantly counteract the extent of liver damage (**F**,**G**). In addition, NTN administered mice decrease NEFA and TG levels compared to HFD mice (**H**,**I**). FITC-dextran permeability of jejunum was very low in SD mice (**J**). Contrarily, after HFD, mice displayed an increased intestinal permeability that was reduced by NTN administration (**J**). Data are representative of at least three independent experiments. Values are means ± SEM. One-Way ANOVA test. *** *p* < 0.001 vs. SD; # *p* < 0.05 vs. HFD; ## *p* < 0.01 vs. HFD; ### *p* < 0.001 vs. HFD.

As demonstrated by several in vivo studies, excess dietary fat induces significant body weight gain [41,42]. These assumptions demonstrate a link among increased fat depots, weight gain, and liver damage. In fact, liver injury probably aggravates the metabolic syndrome, supporting that not only the amount of calories is important in the induction of weight gain or metabolic syndrome, but other factors may be involved as well. Our data showed a substantial increase in body weight of HFD-fed mice compared to the control group (Figure 6A). Two weeks of NTN treatment appreciably reduced weight gain from the first week of treatment (Figure 6A). No significant variations were found in weekly food intake (Figure 6B).

Through H&E staining we evaluated liver tissue integrity. Mice fed with HFD demonstrated an accentuated hydropic degeneration and steatosis that was diffusely distributed throughout all areas of the hepatic acinus (Figure 6E, histological score Figure 6G) compared to control mice (Figure 6C, histological score Figure 6G).

On the other hand, lipid intolerant mice treated with NTN showed a meaningful reduction in hydropic degeneration and steatosis (Figure 6F, histological score Figure 6G).

A physiological increase in non-esterified fatty acids (NEFA) and triglycerides (TG) plasma levels is usually observed after an intake of a high-fat meal [43,44].

Hence, to determine whether the HFD-fed and NTN treated mice differ in their capacity to handle acute lipid challenge, we performed a lipid tolerance test.

A significant increase in circulating NEFA and TG levels was observed in mice fed with HFD compared to SD-fed mice (Figure 6H and Figure 6I respectively). NTN treated mice demonstrated a significantly greater capacity to clear an acute rise in NEFA and TG in response to emulsified lipid infusion compared to untreated mice (Figure 6H and Figure 6I respectively).

Furthermore, as described by Tanaka et al. [45], HFD-derived free fatty acids increase sensitivity to intestinal damage; therefore, we have analyzed the barrier protective properties of NTN by assessing intestinal permeability with a Transelectrical Epithelial Resistance (TEER) test in HFD mice. A marked increase in gut permeability was observed in mice fed with HFD compared to mice fed with an SD (Figure 6J). However, two-week NTN treatment significantly reduced gut permeability (Figure 6J).

### 3.6. Effects of NTN Administration on Intestine Epithelial Integrity in HFD Mice

**Figure 7:**
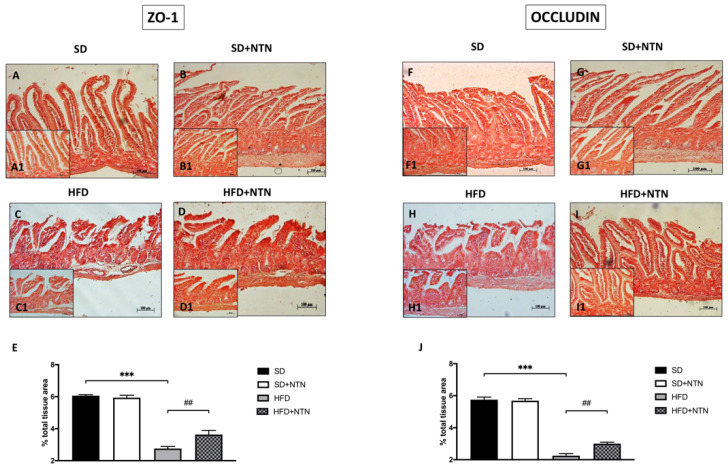
Effects of NTN administration on intestine epithelial integrity in HFD mice. High expressions of ZO-1 and Occludin have been found in intestinal tissues of the SD group and SD + NTN group ((**A**,**B**,**E**) and (**F**,**G**,**J**) respectively) compared to the HFD group ((**C**,**E**) and (**H**,**J**) respectively). The administration of NTN restored the expression of ZO-1 and Occludin proteins ((**D**,**E**) and (**I**,**J**) respectively). Data are representative of at least three independent experiments. Values are means ± SEM. One-Way ANOVA test. *** *p* < 0.001 vs. SD; ## *p* < 0.01 vs. HFD.

We investigated the effect of NTN on ZO-1 and Occludin expressions by immunohistochemical staining also in an HFD model. The obtained results revealed a basal expression of ZO-1 and Occludin in the tissues of the SD group (Figure 7A, histological score Figure 7E and Figure 7F, histological score Figure 7J respectively); while the HFD group was characterized by a reduction of both TJs expression (Figure 7C, histological score Figure 7E and Figure 7H, histological score Figure 7J respectively). NTN treatment was able to appreciably upturn ZO-1 and Occludin expressions (Figure 7D, histological score Figure 7E and Figure 7I, histological score Figure 7J respectively), thus repairing the compromised intestinal permeability.

### 3.7. Effects of NTN Administration on Abdominal Adipose Tissue Damage in HFD Mice

Histopathological analysis of white adipose tissue from the abdomen was performed by H&E staining.

**Figure 8:**
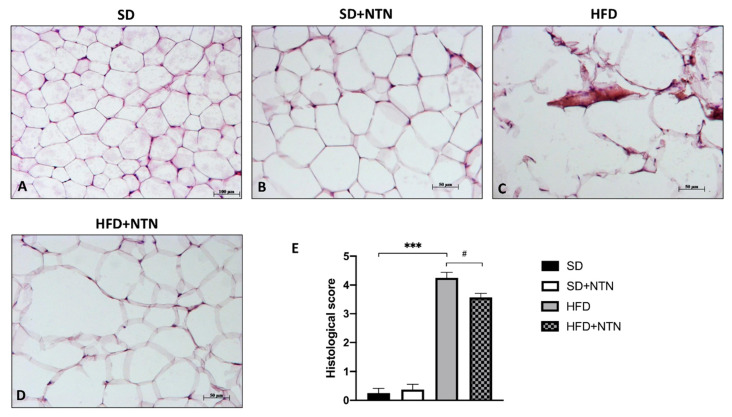
Effects of NTN on adipose damage in HFD mice. HFD led to a remarkable increase in neutrophil infiltration and adipocyte size (**C**,**E**) compared to SD and SD + NTN mice (**A**,**B**,**E**). Administration of NTN was able to significantly counteract the extent of adipose tissue due to HFD (**D**,**E**). Data are representative of at least three independent experiments. Values are means ± SEM. One-Way ANOVA test. *** *p* < 0.001 vs. SD; # *p* < 0.05 vs. HFD.

Lipid intolerant mice displayed a significant increase in the size of adipocytes and neutrophil infiltration (Figure 8C, histological score Figure 8E) compared to control mice (Figure 8A, histological score Figure 8E). Treatment with NTN significantly improved tissue architecture by reducing the adipocytes size as well as the neutrophil infiltration (Figure 8D, histological score Figure 8E).

### 3.8. Effects of NTN Administration on Body Weight, Food Intake and Liver Tissue Damage in HFrD Mice

Although HFrDs have been implicated in obesity via impairment of leptin signaling in humans, several in vivo studies [30,46,47] have invalidated these assumptions in mice.

This could be related to the higher mass-specific metabolic rate of mice, which might allow for greater tolerance to fructose consumption. In fact, the fructose may be oxidized to CO_2_ and H_2_O to a greater extent in mice than rats, without the deleterious effects of fructose metabolites shuttled into VLDL (very low-density lipoprotein) synthesis [47].

**Figure 9:**
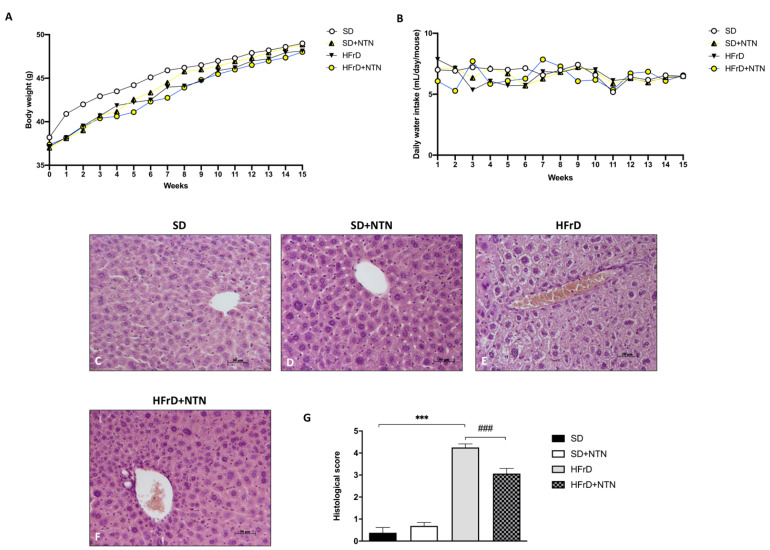
Effects of NTN on liver damage in HFrD. No considerable variations were detected in body weight and food intake in HFrD mice (**A**,**B**). Significant macrovesicular and microvesicular steatosis was observed in mice fed with HFrD (**E**,**G**) compared to SD and SD + NTN mice (**C**,**D**,**G**). The administration of NTN was able to significantly counteract the extent of liver damage in HFrD mice (**F**,**G**). Data are representative of at least three independent experiments. Values are means ± SEM. One-Way ANOVA test. *** *p* < 0.001 vs. SD; ### *p* < 0.001 vs. HFrD.

The results obtained from the analysis of body weight and water intake in HFrD mice did not show significant differences (Figure 9A,B), thus confirming that excessive fructose consumption is not directly related to body weight gain.

To evaluate the effect of NTN on liver tissue damage in fructose intolerant mice, we carried out a histological examination by H&E.

Mice fed with a HFrD significantly increased liver tissue damage (Figure 9E, histological score Figure 9G) compared to control mice (Figure 9C, histological score Figure 9G). NTN significantly reduced chronic inflammation, macrovesicular and microvesicular steatosis following an HFrD diet, improving liver tissue architecture (Figure 9F, histological score Figure 9G).

### 3.9. Effects of NTN Administration on Intestinal Tissue Damage and Permeability in HFrD Mice

Fructose intolerance is often associated with malabsorption and gastrointestinal disorders, including both increased intestinal motility and sensitivity, which overall lead to impaired bowel function [20,48].

Therefore, we executed H&E staining to evaluate the effect of NTN on intestinal tissue damage in fructose intolerant mice.

**Figure 10:**
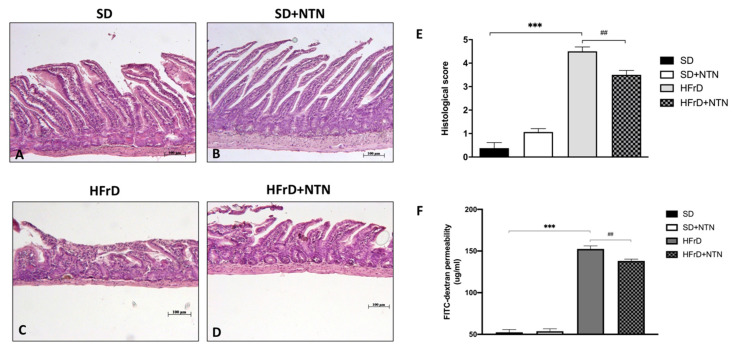
Effects of NTN on intestinal features in HFrD mice. Significant infiltration of inflammatory cells and tissue damage was observed in mice fed with HFrD (**C**,**E**) compared to SD and SD + NTN mice (**A**,**B**,**E**). Administration of NTN was able to significantly counteract the extent of intestinal tissue damage (**D**,**E**). A gut permeability assay exhibited an evident increase of intestinal permeability in HFrD jejunum compared to the SD group (**F**); NTN showed protective properties decreasing gut permeability (**F**). Data are representative of at least three independent experiments. Values are means ± SEM. One-Way ANOVA test. *** *p* < 0.001 vs. SD; ## *p* < 0.01 vs. HFrD.

Mice fed with a HFrD significantly increased intestinal tissue damage, as observed by the loss of the lamina propria structure as well as inflammatory cell infiltration (Figure 10C, histological score Figure 10E) compared to control mice (Figure 10A, histological score Figure 10E).

Two weeks of NTN administration significantly reduced neutrophilic inflammation and edema improving intestinal tissue architecture (Figure 10D, histological score Figure 10E).

Moreover, elevated levels of fructose in the diet result in increased intestinal permeability [30]; thus, to assess the effect of NTN on gut permeability in fructose intolerant mice, we performed a FITC-dextran permeability assay.

A marked increase in gut permeability was observed in mice fed with an HFrD compared to mice fed with an SD (Figure 10F). NTN treatment significantly reduced gut permeability after two weeks of treatment (Figure 10F).

### 3.10. Effect of NTN on Epithelial Integrity in the Intestines of HFrD Mice

Chronic fructose intake is also associated with a loss of tight junction proteins, resulting in dysfunction of the intestinal barrier [49].

In relation to this, we estimated the possible positive outcome of NTN on intestinal epithelial integrity through immunohistochemical localization of ZO-1 and Occludin.

**Figure 11:**
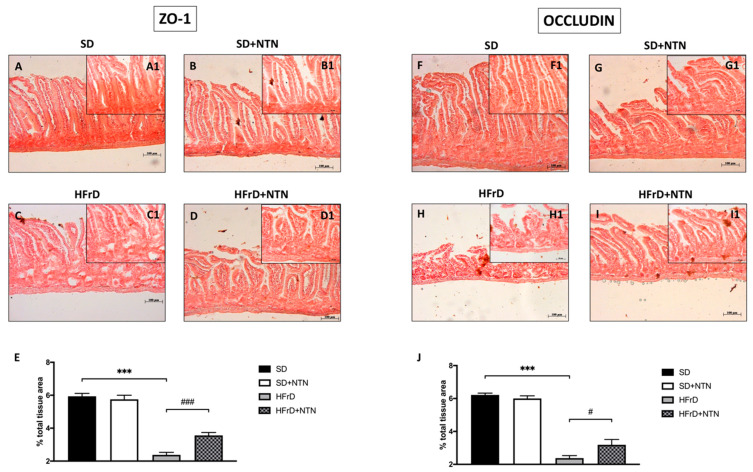
Effect of NTN on intestinal epithelial integrity in HFrD mice. Intestinal tissues of SD and SD + NTN mice displayed high expressions of ZO-1 (**A**,**B**,**E**) and Occludin (**F**,**G**,**J**) proteins, contrariwise TJs expressions were reduced after HFrD (**C**,**E**,**H**,**J**). NTN two-week treatment significantly restored ZO-1 (**D**,**E**) and Occludin (**I**,**J**) expressions. Data are representative of at least three independent experiments. Values are means ± SEM. One-Way ANOVA test. *** *p* < 0.001 vs. SD; # *p* < 0.05 vs. HFrD; ### *p* < 0.001 vs. HFrD.

Mice fed with a HFrD displayed a significant decrease in ZO-1 (Figure 11C, histological score Figure 11E) and Occludin expressions (Figure 11H, histological score Figure 11J) compared to SD-fed mice (Figure 11A and Figure 11F respectively, histological score Figure 11E and Figure 11J respectively).

The two-week treatment with NTN considerably improved the integrity of the intestinal barrier (Figure 11D,I, histological score Figure 11E,J).

## 4. Discussion

FIs refer to the difficulty in digesting certain foods; these disorders afflict a consistent percentage of the population, representing an influencing factor for the development of other pathologies such as irritable bowel syndrome (IBS) etc. [50]. In fact, up to 65% of IBS patients report that their symptoms are related to specific foods, this overlapped clinical sign makes diagnosis even more difficult for FIs patients [51]. However, a sizable percentage of patients show gastrointestinal complaints, similar to indigestion, without a specific diagnosis, making the management of symptoms, in the meanwhile, a priority [52].

It is important to underline that FIs are food disorders quite distinct from food allergies. In fact, in the case of FIs, the immunological component is not implicated; consequently, the individual does not react with an immune response but immediately replies to food ingestion with gastrointestinal and/or extraintestinal symptoms [52]. Although FIs are not life-threatening as in the case with food allergies, they still represent an uncomfortable condition for patients’ quality of life. Following a comprehensive medical history including dietary and lifestyle evaluation, with a focus on potential FIs, patients with gastrointestinal symptoms are usually subjected to clinical examinations which may include blood and fecal tests, endoscopy and/or radiological imaging to rule out any organic disease or food allergy [1]. However, there are a limited number of clinically useful tests available to recognize specific FIs. This unfavorably affects the patient’s quality of life in terms of social activities and reduced dietary choice in order to achieve symptom improvement [1].

Actually, FIs therapeutic options involve the adoption of eating plans that assume the elimination of specific foods from the diet; however, they are difficult to maintain long term and often are not healthy approaches [53].

The discovery and subsequent employment of new therapeutic strategies could confer a new hopeful perspective on FIs.

The restoration of the integrity of the intestinal mucosa would represent a valid support to regulate nutrient sensing, thus helping to relieve FIs symptoms. Perturbation of gut barrier homeostasis can lead to increased epithelial permeability and dysbiosis of the microbiota, which has been recognized as playing a key role in the pathophysiology of several gastrointestinal disorders [54].

In this regard, many researchers highlighted a strong connection between food hypersensitivity and intestinal disruption, suggesting this target as a promising therapeutic solution [55].

In recent years, new scientific findings promoted the effects of probiotics and dietary enzymes to help break down sugars in fructose and lactose intolerant patients [23,56]. Alternative solutions that can reestablish the gut microbiota and promote gut homeostasis regardless of the FIs are needed.

Given these outcomes, we investigated the beneficial effect of a natural-based therapeutic in multiple murine models of FIs.

The intestinal mucosa represents the main tissue to investigate disease-related metabolism [57]. In particular, in the context of FIs, adverse reactions to food may cause the progressive alteration of the intestinal barrier, resulting in the development of a persistent inflammatory condition and impaired intestinal motility, sensitivity and permeability [17,18].

Our results showed that the intake of HCD, HFD and HFrD led to a marked increase in intestinal permeability. Contrarily, treatment with NTN effectively provided rapid symptom relief by restoring the compromised gut permeability in carbohydrate, lipid, and fructose intolerant mice within two weeks of treatment. It is widely known as an intact intestinal barrier is important to prevent the entry of endotoxins, microorganisms and undigested food particles while allowing physiological functions including but not limited to essential nutrients and water absorption to take place [18].

This physical barrier is held together by the TJs, such as Occludin that creates bridges between intracellular zonula occludens. Concerning this, the role of epithelial TJs is crucial to seal off gaps between cells and in maintaining gut homeostasis [58].

Furthermore, several pre-clinical studies reveal that TJs breakdown is typical in many intestinal diseases, including FIs [30].

Our data confirmed, together with an alteration of the intestinal mucosal architecture, a TJ dysregulation due to HCD, HFD and HFrD. Nevertheless, two weeks of NTN treatment is proven to extensively recover intestinal tissue damage and restore Occludin and ZO-1 expressions in carbohydrate, lipid and fructose intolerant mice. These positive outcomes are attributable to the modulation of intestinal bacteria activities and to protective barrier properties exerted on the intestinal mucosa [59,60]; which led to a restoration of the intestinal epithelial barrier.

Interestingly, Do et al. [30] reported how the loss of intestinal permeability precedes lipid accumulation, which is subsequently associated with hepatic steatosis. In relation to this, other evidence supported the close correlation and cooperation between the gut and the liver, defined as the gut-liver axis [61]. In this reciprocal connection, the integrity of the gut barrier plays a fundamental role in maintaining hepatic homeostasis [61].

More specifically, intestinal barrier function loss, due to TJs disruption, allows the passage of pro-inflammatory stimuli such as pathogen-associated molecular patterns (PAMPs) to the liver through the portal system promoting the progression of chronic liver diseases, such as cirrhosis alcoholic liver disease (ALD), and Non-Alcoholic Fatty Liver Disease (NAFLD) [62,63].

The results obtained from this study clearly confirmed an extensive increase in liver fat content following HCD, HFD and HFrD compared to SD. However, two-week NTN administration was able to promote a good recovery in steatosis in HCD mice as well as in counteracting the accumulation of hepatic fat following a hyper lipidic diet, and to moderate the hydropic degeneration of hepatocytes in fructose intolerant mice.

Recent findings [45,64,65] emphasized a crosstalk between intestinal epithelial damage and circulating free fatty acids (FFAs) concentrations. In fact, if on the one hand, the increase in HFD-derived free fatty acids produces “intestinal lipotoxicity”, on the other hand, intestinal function is also involved in the regulation of plasma levels of FFAs [65,66]. This thesis is supported by growing evidence that exposes how the intestine actively participates in the regulation of the lipid metabolism of the whole body through the regulation of nutrients, hormonal, metabolic and neural regulatory pathways [65]. On this basis, we looked at the main clinical markers of health or disease status of dyslipidemic patients, such as NEFA and TG.

Our data visibly revealed an increased concentration of both NEFA and TG in HFD mice. Two week NTN administration showed positive outcomes on lipid intolerance features, thus suggesting a good capacity to handle the lipid load.

Visceral adipose fat (VAT) is a hormonally active tissue and possesses a unique biochemical profile that influences physiological and pathological processes in the human body, including metabolic processes [67]. Visceral obesity is associated with several medical disorders such as metabolic syndrome [68], CVD [69] and a shortened life expectancy [70].

Moreover, several scientific data support how a high daily intake of fatty meals or refined sugars induced a progressive increase in white adipose tissue, especially in the intra-abdominal cavity [71].

Therefore, considering adipose tissue to be a useful biomarker of dietary fatty acid intake and carbohydrate excess consumption as well [72,73], we analyzed its morphological changes.

Consistent with what was previously mentioned, our study exposed how high caloric intake derived from HCD and HFD led to increased body fat, especially in abdominal visceral fat.

De facto, the obtained results showed an expansion in the size of adipocytes together with an increase in neutrophilic infiltration in white abdominal tissue of HCD/HFD mice, while two week NTN treatment appreciably improved the architecture of the abdominal adipose by decreasing adipocytes size and the infiltration of neutrophils in carbohydrate and lipid intolerant mice.

Hyperinsulinemia correlated with hyperglycemia is considered to be a sign of insulin resistance development, which is typical in carbohydrate intolerant patients [74].

Insulin is known to be a key hormone, secreted by β-pancreatic cells, which affects almost all organs in the body, including adipose tissue, liver, and the vascular system as well [75].

In healthy subjects, insulin secretion is coordinated to circadian rhythms, which regulate the daily rhythm in glucose metabolism and whole-body insulin sensitivity [76,77]. Otherwise, reduction in insulin sensitivity as well as its non-physiological fluctuations exposes the tissues to disruption of metabolic molecular pathways including glucose metabolism [78]. High carbohydrate intake also contributes to reduced insulin sensitivity and poor management of glycemic control, thus highlighting the influence of the gastrointestinal tract on glucose metabolism [79].

Hence, considering the vital role of the insulin-glucose feedback loop in sugar blood control, we examined pancreatic tissue integrity and glycemic hallmarks in carbohydrate intolerant mice.

Our results confirmed a substantial increase in glucose-insulin levels and pancreatic islet hyperplasia following an HCD diet. However, two week NTN treatment exerted beneficial properties, improved pancreatic tissue damage, and effectively regulated glycemic parameters in carbohydrate intolerant mice. In this context we speculated that mucomimetic substances were also helpful in the management of carbohydrate intolerance, thus offering a new starting point for additional analyses.

## 5. Conclusions

In conclusion, the data obtained from the present study elucidate the many advantages provided by NTN administration, offering a new approach to FIs management.

The beneficial effects deriving from this new natural-based product have been shown to contribute to the restoration of intestinal mucosal barrier integrity and functionality, thus helping to relieve symptoms related to FIs. These benefits deriving from NTN result in a better management of glycemic dysregulation and lipid load as well as fructose intolerance features.

Therefore, considering these new insights, NTN could represent a promising natural support in the non-pharmacological strategy for patients suffering from FIs and intestinal permeability, improving their social relationships and their quality of life. However, we are aware of the limitations of animal models in the translational reproduction of human metabolic disorders, especially in the field of FIs.

In fact, although rodents’ models replicate many aspects of human metabolic disorders, main dissimilarities between species in basal metabolic rate, feeding behavior, fecundity, immune system, and gut microbiota composition should be considered.

Moreover, animal models are also influenced by the environmental conditions and the genetic background.

In this perspective, future evaluations of NTN in well-designed clinical trials could further deepen our knowledge for patient care in those suffering from one and/or multiple FIs.

## Figures and Tables

**Table 1:** NTN formulation. The table indicates the NTN components and the relative dosage. Doses were converted on the basis of mouse body surface formulation.

INGREDIENTS	QUANTITY (mg)
*Acacia senegal* (L.) Willd. (gummi)	100
*L. acidophilus* tyndalized	10
*L. reuteri* tyndalized	7
Pea protein	50
Grape seed extract	50
β-galactosidase	13

**Table 2:** Macronutrient composition of high-carbohydrate diet (HCD).

Weight Content (g/kg)	HCD
Milk proteins	140.0
Starch	622.4
Sucrose	100.3
Soy Oil	40.0
Minerals	35.0
Vitamins	10.0
Cellulose	50.0
Choline	2.3
**Energy Content** (**%**)	**HCD**
Protein	14.7
Carbohydrate	75.9
Fat	9.4
Energy density (kJ/g)	15.95
Food quotient	0.946

## Data Availability

All the results were generated and included in this study.
