# Correction: Ardizzone et al. Efficacy of a Novel Therapeutic, Based on Natural Ingredients and Probiotics, in a Murine Model of Multiple Food Intolerance and Maldigestion. *Nutrients* 2022, *14*, 2251

**DOI:** 10.3390/nu16234172

**Published:** 2024-11-30

**Authors:** Alessio Ardizzone, Marika Lanza, Giovanna Casili, Michela Campolo, Irene Paterniti, Salvatore Cuzzocrea, Emanuela Esposito

**Affiliations:** Department of Chemical, Biological, Pharmaceutical and Environmental Sciences, University of Messina, Viale Ferdinando Stagno D’Alcontres, 98166 Messina, Italy; aleardizzone@unime.it (A.A.); mlanza@unime.it (M.L.); gcasili@unime.it (G.C.); campolom@unime.it (M.C.); ipaterniti@unime.it (I.P.); salvator@unime.it (S.C.)

In the original publication [1], there was a mistake in Figure 7F as published. The corrected Figure 7 appears below.

The authors state that the scientific conclusions are unaffected. This correction was approved by the Academic Editor. The original publication has also been updated.

## Figures and Tables

**Figure 7:**
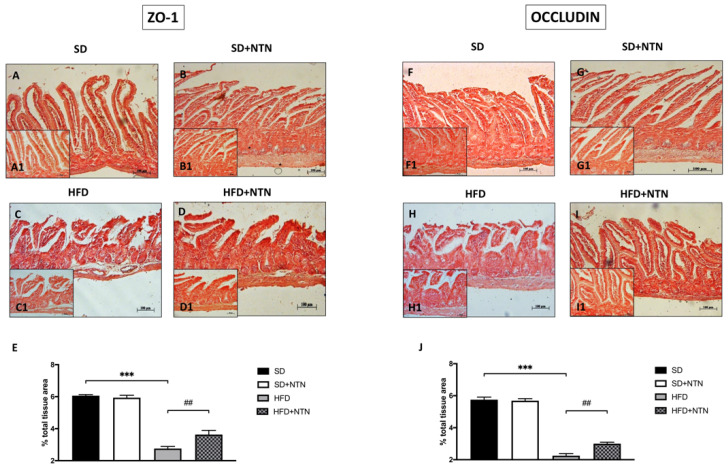
Effects of NTN administration on intestine epithelial integrity in HFD mice. High expressions of ZO-1 and Occludin have been found in intestinal tissues of the SD group and SD + NTN group ((**A**,**B**,**E**) and (**F**,**G**,**J**) respectively) compared to the HFD group ((**C**,**E**) and (**H**,**J**) respectively). The administration of NTN restored the expression of ZO-1 and Occludin proteins ((**D**,**E**) and (**I**,**J**) respectively). Data are representative of at least three independent experiments. Values are means ± SEM. One-Way ANOVA test. *** *p* < 0.001 vs. SD; ## *p* < 0.01 vs. HFD.
